# Neoadjuvant chemotherapy followed by D2 gastrectomy and esophagojejunal Roux-en-Y anastomosis in gastric small cell carcinoma: A case report

**DOI:** 10.3892/ol.2014.2557

**Published:** 2014-09-24

**Authors:** KAI XIN, JIA WEI, HAO WANG, WENXIAN GUAN, BAORUI LIU

**Affiliations:** 1 The Comprehensive Cancer Center of Nanjing Drum-Tower Hospital, Clinical College of Nanjing Medical University, Nanjing, Jiangsu 210008, P.R. China; 2 Department of General Surgery, Nanjing Drum-Tower Hospital, The Affiliated Hospital of Nanjing University Medical School, Nanjing, Jiangsu 210008, P.R. China

**Keywords:** neoadjuvant chemotherapy, gastric small cell carcinoma

## Abstract

A 60-year-old male was referred to Nanjing Drum-Tower Hospital (Nanjing, Jiangsu, China) due to the presence of gastric carcinoma. A biopsy was performed under an electronic gastroscope and the pathological analysis resulted in the diagnosis of gastric small cell carcinoma (GSCC). The mass had invaded the liver and the pancreas according to an enhanced computed tomography scan, thus current surgical methods were considered to be of high risk and highly challenging. Following four cycles of neoadjuvant chemotherapy with irinotecan (200 mg, days 1, 21, 41 and 61) and oxaliplatin (120 mg, days, 1, 21, 41 and 61) the patient underwent a D2 gastrectomy and an esophagojejunal Roux-en-Y anastomosis, followed by adjuvant chemotherapy. The patient experienced survival without progression in the 8-month follow-up. To the best of our knowledge, this is one of few cases of GSCC treated with the combination of neoadjuvant chemotherapy, surgery and adjuvant chemotherapy.

## Introduction

Small cell carcinomas (SCC) are a group of neuroendocrine tumors, the majority of which arise from the lung (1). When compared with the most frequently reported small cell lung carcinomas (SCLCs), SCCs rarely occur in extra-pulmonary organs and it has been reported that extra pulmonary small cell carcinomas (ESCCs), occuring in urinary, digestive and female reproductive system, account for only a small amount of all SCCs (2).

Gastric SCC (GSCC), one of the typical SCC arising from the digestive system, is a rare small cell neuroendocrine tumor (3). However, GSCC is one of the most aggressive malignant tumors and has an extremely poor prognosis due to its high metastatic rate, with atypical clinical manifestations (4). There are few studies of cases of GSCC (5–9), with great variability in how the tumors are treated, and little clear guidance is currently available (10). The present study reports the case of a 60-year-old male diagnosed with GSCC who received a combined treatment of neoadjuvant chemotherapy, surgery and adjuvant chemotherapy. Written informed consent was obtained from the patient.

## Case report

**Figure 1:**
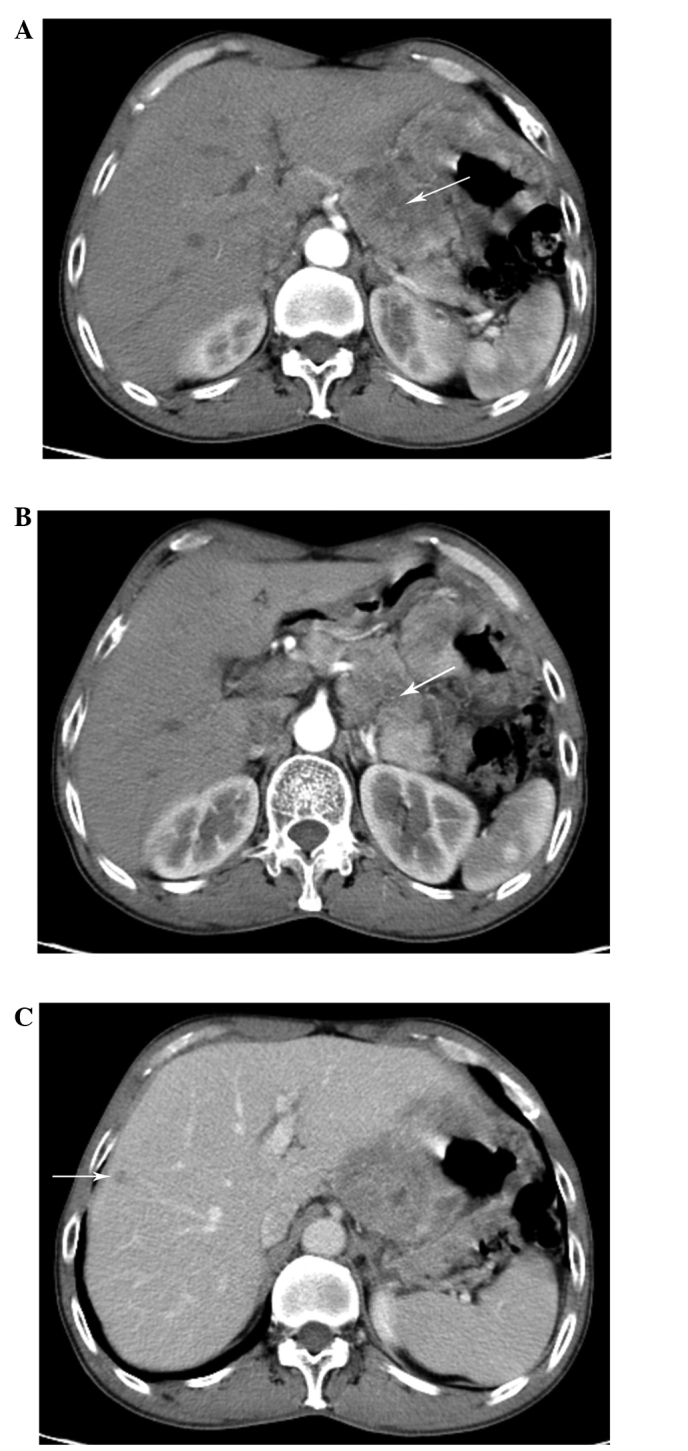
Enhanced computed tomography prior to the neoadjuvant chemotherapy. (A) A mass (arrow) in the gastric wall. (B) The mass (arrow) invading the liver and pancreas. (C) A nodular shadow (arrow) of low density, with mild enhancement.

A 60-year-old male presented to the Nanjing Drum-Tower Hospital (Nanjing, Jiangsu, China) with a one-year history of epigastric distress. No other symptoms, including acid regurgitation, eructation, nausea, emesis, diarrhea or melena, were described. The patient had no history of hypertension or diabetes mellitus and suffered from no infectious diseases, such as hepatitis or tuberculosis. The patient had smoked one pack of cigarettes per day for the past 30 years and had consumed alcohol for ~20 years, but had no significant drug use or history of malignancy in first-degree relatives. There was no evident histological abnormality upon physical examination. An electronic endoscopy revealed irregular tumor-like lesions in the cardia, lesser curvature of the stomach and angular notch. A separate, large, ulcerated mass with friable mucosa was observed in the gastric cardia. Multiple biopsy specimens were obtained. The hematoxylin and eosin-stained sections revealed densely-packed sheets of small basophilic cells. Immunostaining was positive for cytokeratin and the tumor was focally positive for synaptophysin and chromogranin A. The immunohistochemical profile supported the histological diagnosis of an SCC. No abnormality was detected on chest radiography. A routine blood examination, liver function test and electrocardiogram were performed. Among all the tumor markers, the level of carbohydrate antigen (CA)-125 was 69.80 U/ml, slightly higher than the normal range of 0–35 U/ml. Computed tomography (CT) revealed a thickening of the gastric wall and a mass (Fig. 1A), mainly in the lesser curvature of stomach. A thickening left gastric artery entered the mass. The mass invaded the liver and the pancreas (Fig. 1B), and the enhanced CT scan revealed a nodular shadow of low density, with mild enhancement (Fig. 1C).

**Figure 2:**
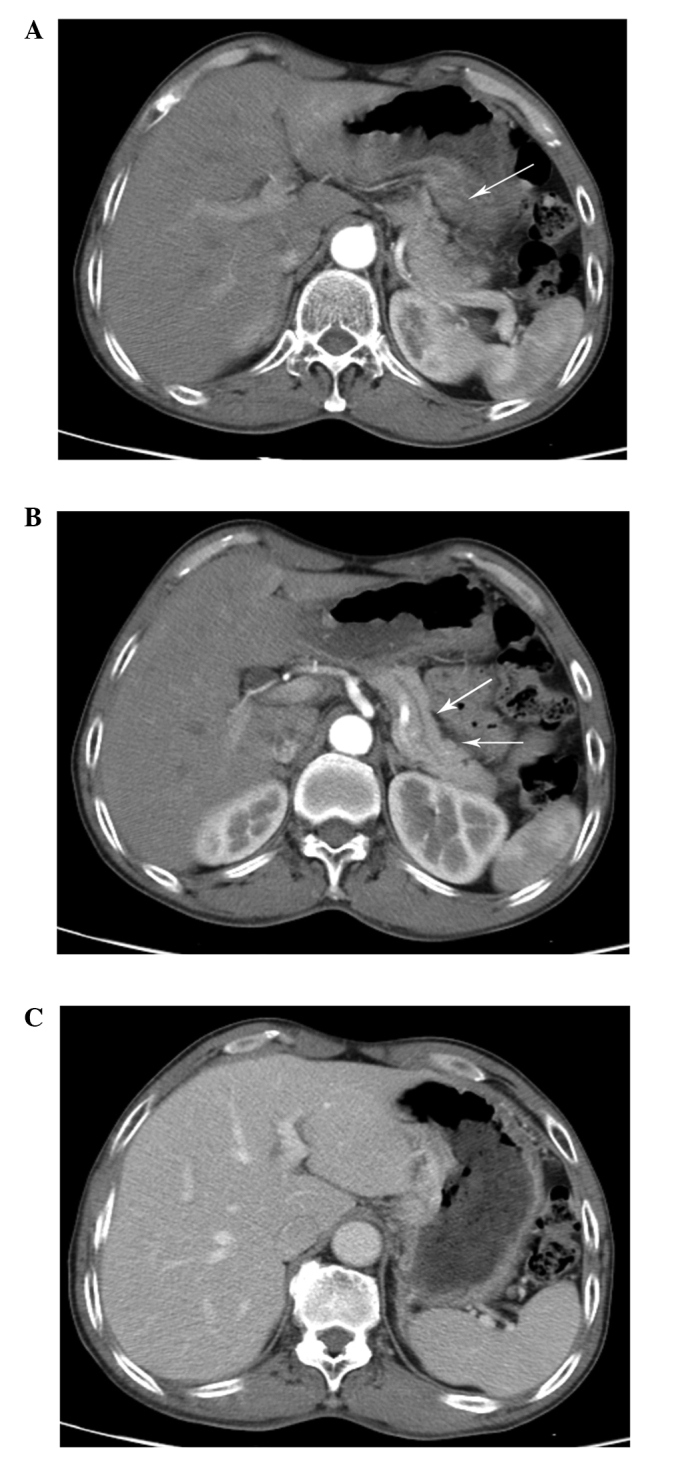
(A and B) Enhanced computed tomography following two cycles of neoadjuvant chemotherapy revealing that the lesion (arrows) was clearly decreased in size. (C) There is no evident nodule in the liver.

**Figure 3:**
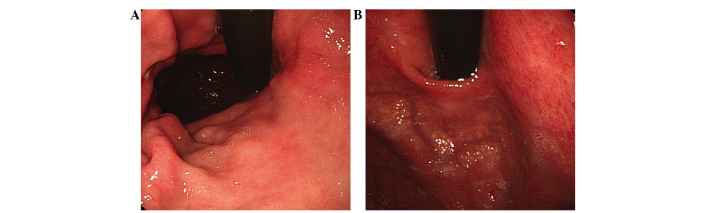
Electronic gastroscopy, showing the few nodular niduses in the mucosa of the (A) gastric body and (B) the fundus of the stomach.

Collectively, the electronic gastroscopy (EG) and CT findings supported a final diagnosis of metastatic GSCC. Following a multi-disciplinary discussion between the Departments of General Surgery and Radiation Oncology, it was decided that current surgical methods would be of high risk and highly challenging. A plan was developed to start the treatment of the patient using neoadjuvant chemotherapy. Therefore, the patient underwent treatment with irinotecan (200 mg, days 1, 21, 41 and 61) and oxaliplatin (120 mg, days 1, 21, 41 and 61) for a four-cycle period, without an evident adverse reaction. Following two cycles of chemotherapy, the patient achieved a partial response. The CT scan revealed that the lesion had clearly decreased in size (Fig. 2A and B). The enhanced CT scan revealed that there was no evident nodule in the liver (Fig. 2C). Levels of the CA-125 serum tumor marker dropped to within the normal range. Following four cycles, the patient achieved another partial response, and then another EG was performed. There were certain nodular niduses in the gastric body and the fundus of the stomach (Fig. 3A and B). No abnormality was found in the mucosa of the gastric antrum, neither were congestion, edema, ulcers, bleeding or any other symptoms.

**Figure 4:**
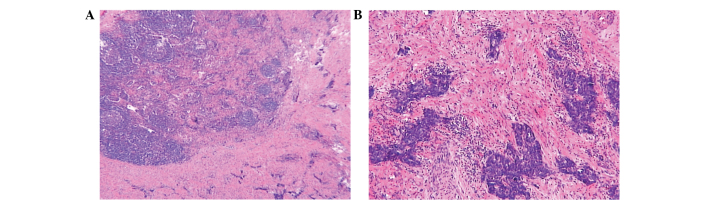
(A and B) Post-surgical pathology of the mass revealing small-cell carcinoma of the stomach. (A) Magnification, ×100. (B) Magnification, ×200.

Following another multi-disciplinary discussion, the patient underwent a D2 gastrectomy and esophagojejunal Roux-en-Y anastomosis. There was a 5×2×1-cm mass under the cardia of the stomach, in the lesser curvature. The serous membrane layer was complete and there were no metastatic nodules found in the liver, pancreas, spleen, kidneys, abdominal wall or pelvic cavity. The post-surgical pathology revealed that the tumor was 4×4×0.8 cm in size, invading the serosa, nerves and vessels of the stomach. The surgical margin was revealed to be tumor-free. Three metastasis-positive lymph nodes were found in 27 cleaned lymph nodes around the stomach. The pathological tumor-node-metastasis stage was stage IV (T4aN2M1; Fig. 4A and B). Following surgery, an enhanced CT scan was performed and the patient received four cycles of adjuvant chemotherapy combining irinotecan (200 mg, days 1, 21, 41 and 61) and oxaliplatin (120 mg, days 1, 21, 41 and 61). The patient was followed up for ~8 months and is currently alive.

## Discussion

The neuroendocrine tumors of the digestive system are typically classified by their differentiation according to the World Health Organization classification (11) as follows: Well-differentiated, subdivided into benign and borderline tumors; moderately-differentiated, with low malignant potential; poorly-differentiated, including large cell neuroendocrine carcinoma and SSC; or mixed gland/neuroendocrine carcinoma (12,13). SCCs are a group of neuroendocrine tumors. SCCs are extremely rare, with the majority occurring in the lung. Extrapulmonary SCC (EPSCC), which occurs in the bladder, prostate, esophagus, stomach, colon, rectum and gallbladder, has been reported domestically in China and overseas (14). EPSCC is rare and accounts for 4% of the total number of SCCs. However, GSCC is extremely rare and accounts for ~0.1% of all EPSCCs. In addition, according to the literature, GSCC accounts for ~11% of all gastrointestinal SCCs (10) and ~0.1% of all gastric cancers.

SCC of the stomach is rarely reported. The condition was first reported in 1976 by Matsusaka *et al* (17). GSCCs are large solid tumors, with histological features similar to those of small-cell lung carcinoma. The majority of GSCCs arise in the upper one-third of the stomach (8,18) and tend to exhibit highly aggressive biological behavior, with a tendency for early distant metastases. There are reports of GSCC cases in China (19–21) and, notably, Huang *et al* summarized and analyzed the clinical features and prognosis of GSCC (22). However, the methods of GSCC therapy are rarely mentioned in these studies. The present study reports one of the extremely few cases of neoadjuvant chemotherapy in GSCC patients. Chemotherapy has less effect on GSCC compared with small cell lung cancer (SCLC) (6). GSCC has previously been treated with regimens for SCLC, with the combination of etoposide and cisplatin. Phase II/III studies of irinotecan combined with cisplatin in patients with SCLC have demonstrated the usefulness of irinotecan/carboplatin in the chemotherapy for SCLC (18–20). Oxaliplatin, the third generation of the platinum drugs, has a reduced gastrointestinal reaction compared with that of cisplatin.

In the present study, following the pathological diagnosis of GSCC, the enhanced CT scan revealed that the mass had invaded the liver and the pancreas, with a nodular shadow of low density in the liver and mild enhancement. Current surgical methods were concluded to be of high risk and highly challenging following multi-disciplinary discussion. Subsequent to four cycles of neoadjuvant chemotherapy with irinotecan and oxaliplatin, the enhanced CT scan revealed that the lesion was markedly decreased in size and that there was no clear nodule in the liver. The patient then received a D2 gastrectomy and esophagojejunal Roux-en-Y anastomosis, followed by adjuvant chemotherapy. The present case of combined neoadjuvant chemotherapy, surgery and adjuvant chemotherapy highlights the comprehensive treatment of GSCCs, particularly neoadjuvant chemotherapy, which provided the patient with an opportunity to receive surgery. The present patient is currently alive without disease progression and the follow-up of this case continues.
